# Terrestrial capture of prey by the reedfish, a model species for stem tetrapods

**DOI:** 10.1002/ece3.2694

**Published:** 2017-04-21

**Authors:** Sam Van Wassenbergh, Christoffel Bonte, Krijn B. Michel

**Affiliations:** ^1^ Department of BiologyUniversity of AntwerpAntwerpBelgium; ^2^ Département d'Ecologie et de Gestion de la BiodiversitéMuséum National D'Histoire NaturelleUMR 7179 CNRSParis Cedex 05France; ^3^ Structure & Motion LaboratoryThe Royal Veterinary CollegeHatfieldHertfordshireUK

**Keywords:** feeding, polypteridae, prey‐capture, terrestrialization

## Abstract

Due to morphological resemblance, polypterid fishes are used as extant analogues of Late Devonian lobe‐finned sarcopterygians to identify the features that allowed the evolution of a terrestrial lifestyle in early tetrapods. Previous studies using polypterids showed how terrestrial locomotion capacity can develop, and how air ventilation for breathing was possible in extinct tetrapodomorphs. Interestingly, one polypterid species, the reedfish *Erpetoichthys calabaricus*, has been noted being capable of capturing prey on land. We now identified the mechanism of terrestrial prey‐capture in reedfish. We showed that this species uses a lifted trunk and downward inclined head to capture ground‐based prey, remarkably similar to the mechanism described earlier for eel‐catfish. Reedfish similarly use the ground support and flexibility of their elongated body to realize the trunk elevation and dorsoventral flexion of the anterior trunk region, without a role for the pectoral fins. However, curving of the body to lift the trunk may not have been an option for the Devonian tetrapodomorphs as they are significantly less elongated than reedfish and eel‐catfish. This would imply that, in contrast to the eel‐like extant species, evolution of the capacity to capture prey on land in early tetrapods may be linked to the evolution of the pectoral system to lift the anterior part of the body.

## 1 Introduction

The origin of tetrapods and their invasion of terrestrial environments was a major event in vertebrate evolution (Ashley‐Ross, Hsieh, Gibb, & Blob, 2013). The transition from Late Devonian lobe‐finned fishes such as *Eusthenopteron*,* Panderichthys*, and *Tiktaalik* to early tetrapods such as *Acanthostega* and *Ventastega* shows that the origin of tetrapods involved a multitude of morphological changes to the locomotor, respiratory, sensory, and feeding systems (Ahlberg & Milner, 1994; Coates & Clack, 1995; Daeschler, Shubin, & Jenkins, 2006; Jarvik, 1980; Laurin, 2010; Shubin, Daeschler, & Jenkins, 2006). However, as the link between form and function is often not evident from these fossils alone (Ashley‐Ross et al., 2013), modern model species have an important role in identifying the functional implication of the morphological changes in this Late Devonian period (Brainerd, Liem, & Samper, 1989; Ijspeert, Crespi, Ryczko, & Cabelguen, 2007; Markey & Marshall, 2007; Standen, Du, & Larsson, 2014).

Extant polypterids fishes are important models for fishapods. They have retained many of the features of the common ancestor of actinopterygians and sarcopterygians and therefore occupy the most basal position in the actinopterygian phylogeny (Inoue, Miya, Tsukamoto, & Nishida, 2003; Near et al., 2012). Polypteridae possess several morphological traits which are comparable to the Late Devonian lobe‐finned fishes (Elpistostegalia): a relatively elongate cylindrical body form, rhomboid scales that interlock with peg‐and‐socket articulations, ventrolaterally positioned pectoral fins, functional lungs, and similar overall shape and suture morphology of the skull (Claeson, Bemis, & Hagadorn, 2007; Markey & Marshall, 2007; Standen et al., 2014). A recent experimental study relied on similarities in the locomotor system between polypterids and these stem tetrapods to infer the locomotor efficacy on land in the earliest tetrapods (Standen et al., 2014). An earlier study used *Polypterus* to demonstrate how ventilation of the lungs without moveable ribs or a diaphragm could allow early tetrapods to breathe actively (Brainerd et al., 1989).

An equally important step in the terrestrialization of sarcopterygians is the evolution of terrestrial feeding. Early tetrapods were confronted with serious constraints on terrestrial foraging due to density and viscosity differences between water and air (Herrel, Van Wassenbergh, & Aerts, 2012). To infer which of the stem tetrapods had a feeding behavior similar to *Polypterus* (an aquatic suction feeder; Lauder, 1980), and to determine which species were more likely to perform terrestrial biting, Markey and Marshall (2007) compared the cranial suture morphology of *Polypterus* to that of early lobe‐finned fishes and tetrapods. Suture resemblance with *Polypterus* was still present in *Eusthenopteron* but lost in the later fossils *Acanthostega* and *Phonerpeton* (Markey & Marshall, 2007). This is suggestive of a terrestrial feeding mode in the latter two taxa. This study illustrates the central importance of polypterid fishes in reconstructing the evolution of early tetrapods, but does not explain which changes are needed to other cranial components or postcranial anatomy to make the transition from a suction feeder to terrestrial feeder (Michel, Heiss, Aerts, & Van Wassenbergh, 2015; Van Wassenbergh et al., 2006).

Interestingly, in addition to being a suction feeder in water, the polypterid *Erpetoichthys calabaricus* or reedfish has been noted being capable of capturing prey in the terrestrial environment (Sacca & Burggren, 1982). This amphibious fish can survive on land and perform terrestrial locomotion with its elongated body (Pace & Gibb, 2011), making it a prime example of a vertebrate living at the transition between the aquatic and terrestrial environment. It was observed that reedfish regularly left the water and consumed terrestrial insects within a laboratory setting (Sacca & Burggren, 1982). However, it remains unknown how these fish manage to capture prey on land. In this study, we investigate the general behavior and main kinematic features of terrestrial prey‐capture in the reedfish. Knowing this mechanism will then allow us to identify the key morphological and behavioral characteristics likely to be associated with the evolution of terrestrial food‐capture in stem tetrapods.

## 2 Materials and Methods

**Figure 1:**
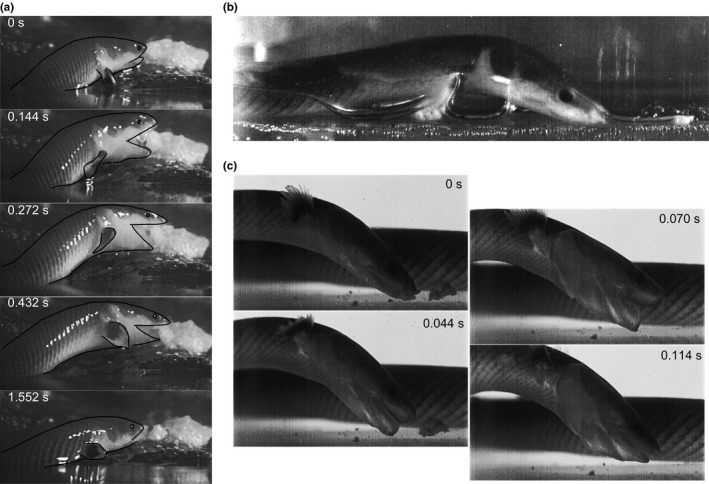
Selected frames from high‐speed videos of prey‐capture by reedfish. (a) Image sequence of terrestrial prey‐capture. Contour lines of the head and pectoral fin were added to improve clarity. A description of this behavior is given in the text. (b) Typical, inclined head posture during terrestrial searches for prey on land. (c) Image sequence of aquatic suction feeding

Six individuals of adult *E. calabaricus* (head length = 19.0 ± 1.6 mm) were acquired from commercial pet trade. Two specimens were kept in a large aquarium with a wooden perch above the water level and were fed pieces of Atlantic cod fillets (*Gadus morhua*). The other four individuals were kept in a two‐compartment aquarium connected by a horizontal terrestrial area that can be reached by surfaces inclined at 45 degrees on both sides (see figure 1 in Van Wassenbergh, 2013). All specimens were kept and handled in accordance with University of Antwerp Animal Care protocols.

High‐speed videos (125 frames per second; Redlake Motionscope M3; IDT, Tallahassee, USA) were recorded during terrestrial capture of prey (two individuals). High‐speed videos at 500 frames per second were recorded for benthic, aquatic capture of prey (four individuals). As the animals displayed more activity in low‐light conditions and at night, we used infrared‐LED panels for lighting. Out of the daily recording session during 4 months, we managed to record two feeding events with sufficient image sharpness and contrast from a lateral view on the head, four from a posterior–dorsal view on the head, and a large number of terrestrial excursions and searches for prey. In the aquatic environment, thirteen feeding events were recorded and analyzed from a lateral view.

The length of the head relative to the body (i.e., head length divided by total length) was measured on preserved specimens for *E. calabaricus* (*N *=* *6) and for the other terrestrially feeding fish *Anableps anableps* (*N *=* *3; specimens from Michel, Aerts, Gibb, & Van Wassenbergh, 2015) and *Periophthalmus barbarus* (*N *=* *4; specimens from Michel, Heiss, et al., 2015) and measured on anatomy drawings for the eel‐catfishes *Channallabes apus* and *Gymnallabes typus* (Cabuy, Adriaens, Verraes, & Teugels, 1999; Devaere, Adriaens, Verraes, & Teugels, 2001). Head length was measured as the distance between the anterior tip of the jaws and the posterior margin of the opercle. These ratios were also determined for a broad taxonomic sample of Actinopterygii by measurements on the lateral‐view contour drawings of the fishes from this group from Nelson's (1994) *Fishes of the World* encyclopedia (*N *=* *375). Anguilliform taxa displayed in this book with a folded tail were replaced by measurements on pictures from the scientific literature. These data were compared to the ratios from reconstructions of four Upper Devonian fossils from the stem tetrapod lineage that are sufficiently complete to allow this quantification: *Gooloogongia loomesi* (Johanson & Ahlberg, 1998), *Eusthenopteron* (Ahlberg & Milner, 1994), *Ichthyostega*, and *Acanthostega* (Ahlberg, Clack, & Blom, 2005).

## 3 Results and Discussion

We will describe the kinematic events during terrestrial prey‐capture based on the image sequence shown in Figure 1a (Movie S1). Initially, *E. calabaricus* emerges from the water and propels itself on to the shore, with its head slightly pitched downward (Figure 1a; *t* = 0 s). Once the snout makes contact with the prey, the mouth is opened, and this elevates the skull to some extent as the mandible pushes against the wooden ground surface (Figure 1a; *t* = 0.144 s). The pectoral fins are moved backward to become adjacent to the body, seemingly without making contact with the ground. The body is then propelled forward toward the prey, the anterior part of the trunk is lifted, and the head increases its nose‐down tilting angle (Figure 1a; *t* = 0.272 s). Next, the jaws are closed over the prey, and the lifted pectoral region falls back down to the substrate (Figure 1a; *t* = 0.432 s). The prey is held between the jaws while the reedfish moves back into the water. The dorsoposterior views on the head show that the opercular slits open to release air during terrestrial prey‐captures. While searching for prey in the terrestrial environment, the head angle is often similarly tilted downward to about 20° (Figure 1b). Terrestrial swallowing of prey was not observed: Reedfish always returned to the water for intraoral transport of the prey.

This mode of prey‐capture is remarkably similar to that described for the eel‐catfish *C. apus* (Van Wassenbergh, 2013; Van Wassenbergh et al., 2006). The eel‐catfish also used a lifted trunk and downward inclined head to capture ground‐based prey. Buccal expansion and compression during mouth opening also resulted in air‐bubble release through the opercular slits. Reedfish thus similarly use the ground support and flexibility of an eel‐like body to perform the trunk elevation and dorsoventral flexion of the anterior trunk region. No role for the pectoral fins (not present in adult eel‐catfish) during prey‐capture could be identified in the reedfish. The only notable difference with the eel‐catfish is that the mouth of the reedfish is not open the entire time it is close to food on land, but opened after contact with the prey (Figure 1a). These findings support the hypothesis that body elongation combined with sufficient anterior trunk flexibility enables ancestrally aquatic species to capture prey on land (Van Wassenbergh et al., 2006).

Also aquatic, benthic feeding behavior of the reedfish resembles that of the eel‐catfish (Van Wassenbergh, 2013; see Movie S2; Figure 1c). The reedfish also uses inertial suction feeding, as was previously described by Lauder (1980) for *Polypterus senegalus*. We consistently observed the head being inclined to a relatively steep angle with respect to the substrate during feeding (mean ± *SD*;* N *=* *13; 51 ± 28°; Figure 1c). Skin folds along the upper and lower jaws help to occlude the corners of the mouth during suction in both eel‐catfish and reedfish. This is assumed to be an adaptation for suction feeding, as this extends the distance in front of the mouth at which suction is effective (Muller & Osse, 1984; Skorczewski, Cheer, & Wainwright, 2012; Van Wassenbergh & Heiss, 2016). These skin folds of reedfish appear considerably looser and are stretched more medially by the flow of water (Figure 1c) than those of eel‐catfish.

Recent studies have shown a variety of ways ray‐finned fish can capture prey on land. Mudskippers (*P. barbarus*) pivot about the pectoral fins to bring their mouth toward terrestrial prey (Michel et al., 2014), of which the capture and intraoral transport is often aided by movement of the water retained in the buccal cavity while on land (Michel, Heiss, et al., 2015). The largescale foureyes (*A. anableps*) uses jaw rotation and protrusion to orient the oral jaws downward (Michel, Aerts, et al., 2015). Both mudskippers and four‐eyed fishes make use of their protrusible upper jaws while feeding on land. As this is a derived feature within actinopterygians and not present in reedfish (nor in eel‐catfish), the reedfish's and eel‐catfish's jaw system more closely resembles that of the stem tetrapods. Consequently, from the variety of terrestrial prey‐capture strategies displayed by extant fish, the simplicity of the oral jaw system shared with early tetrapods makes it likely that a similar behavior forms the basis of the terrestrialization of the feeding system in early tetrapods. Except for the case of specialized jaws of *A. anableps* that move downward to pick up food, a lifted pectoral region is a consistent factor in extant ray‐finned fishes during capturing of prey on land.

**Figure 2:**
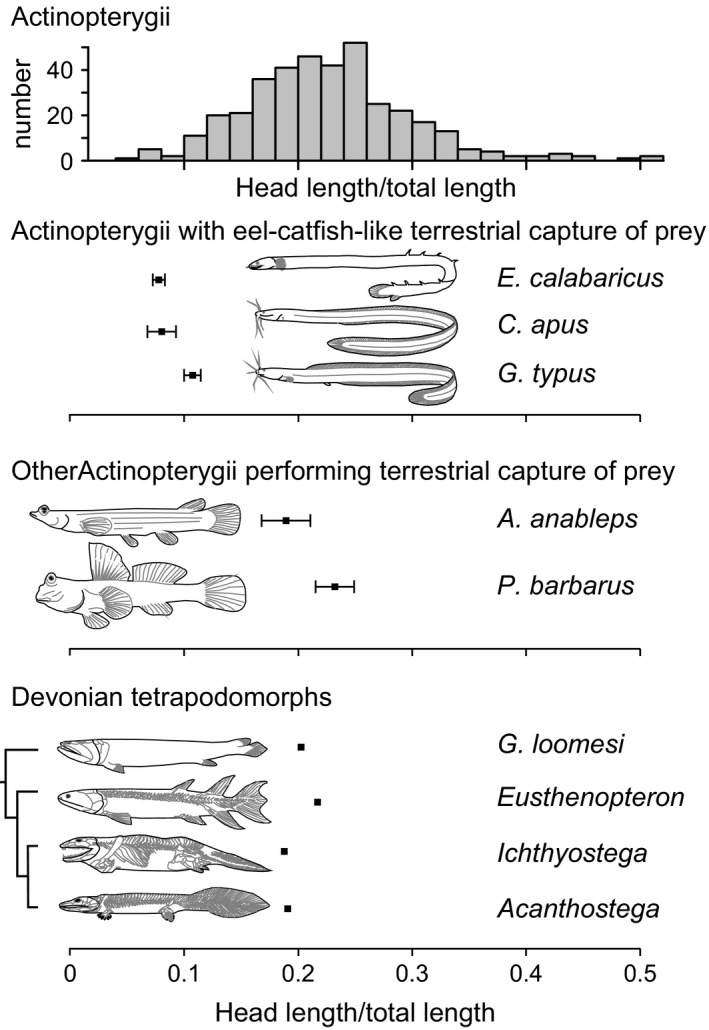
Comparison of head length‐to‐total length ratios between a broad taxonomic sample of ray‐finned fishes (upper histogram; data from Nelson, 1994), the three anguilliform species that terrestrially capture prey (reedfish *Erpetoichthys calabaricus*, eel‐catfish species *Channallabes apus* and *Gymnallabes typus*), and two other terrestrially feeding fish (mudskipper *P. barbarus*, and four‐eyed fish *Anableps anableps*) with four upper Devonian tetrapodomorph fossils (simplified cladogram shown on the left). The four tetrapodomorphs are *G. loomesi* (Johanson & Ahlberg, 1998), *Eusthenopteron* (Ahlberg & Milner, 1994), *Ichthyostega*, and *Acanthostega* (Ahlberg et al., 2005). Note that these tetrapodomorphs are less elongated than reedfish and eel‐catfish, and more closely resemble the average actinopterygian. Error bars denote standard deviation. Numerical data are included as Supporting information (Tables S1–S3)

However, while the reedfish's and eel‐catfish's elongated body and tail allow a controlled and stable lifting of the trunk by curling their anterior part of the body, the stem tetrapods may lack the body length needed to successfully lift the trunk in the same way (i.e., without making use of their pectoral fins or limbs) without losing stability against swaying or rolling sideways. Our analysis of the head length‐to‐total length ratios showed that the stem tetrapods have values between 0.190 and 0.216 (Figure 2). While this is smaller (i.e., more anguilliform) than the average actinopterygian from our broad taxonomic sample (HL/TL = 0.227), values for the reedfish and eel‐catfish are considerably smaller: between 0.078 and 0.107. The analyzed tetrapodomorphs more closely resemble mudskippers (mean ± *SD*; 0.232 ± 0.017) and four‐eyed fishes (0.19 ± 0.02) in this respect. If we assume from this that the capacity to lift the trunk without needing pectoral fins or limbs is unlikely to work in stem tetrapods because of their relatively short body and tail with respect to the anguilliform fishes, development of weight‐bearing capacity by the pectorals is probably essential for the evolution of terrestrial capturing of prey. This would mean that the selective pressure to exploit ground‐based terrestrial prey may have been an important factor in the evolution of pectoral fins to limbs in the earliest tetrapods.

Although the pronounced elongatedness of the reedfish and eel‐catfish may make these species less suitable as model species to infer the capacity and mechanics of terrestrial feeding in tetrapodomorphs, other early tetrapod taxa do show similar or even higher levels of elongatedness. Aistopoda, a group of limbless lepospondyls that lived from early Carboniferous to Early Permian, are extremely elongated (Baird, 1964; Clack, 2012; Germain, 2008). Based on fossils of different species all found in coal swamp localities, the function of the feeding system of aistopods is still debated: Snake‐like cranial kinesis was first proposed (Lund, 1978), but later rejected (Anderson, 2002). Our current data suggest that in analogy with reedfish and eel‐catfish, aistopods probably were capable of capturing relatively small prey on land without the need for a specialized kinesis in their cranial skeletal system. Similarly, a terrestrial prey‐capturing capacity was probably also present in adelogyrinids, another group of elongated lepospondyls, which are presumed to be aquatic suction feeders because they have a highly elaborated hyoid apparatus (Carroll, 1989; Clack, 2012). In reedfish and eel‐catfish, aquatic suction is indispensable to swallow prey that were caught on land. Foraging by adelogyrinids could thus also have taken place at the interface between water and land.

## Conflict of Interest

None declared.
