# Supplementary material for: Terrestrial capture of prey by the reedfish, a model species for stem tetrapods
Source: Ecol Evol. 2017 Apr 21;7(11):3856–60. doi: 10.1002/ece3.2694 (PMC5468123; doi:10.1002/ece3.2694)
Supplement: Supplementary file 1 [file ECE3-7-3856-s001.docx]

**Supplementary Table S1:** List of the head length (HL) to total length (TL) ratios for a broad taxonomic range of Actinopterygii as displayed in Figure 2 (top histogram), and their literature sources.

| **taxon or species name** | **HL / TL** | **Reference** | **page in Nelson 1994** |
| --- | --- | --- | --- |
| *Polypterus* | 0.195 | 1 | 58 |
| Palaeniscoidei | 0.215 | 1 | 59 |
| Acipenseridae | 0.220 | 1 | 60 |
| Polyodontidae | 0.514 | 1 | 61 |
| Dapediinae | 0.251 | 1 | 63 |
| Lepisosteidae | 0.296 | 1 | 63 |
| *Amia calva* | 0.249 | 1 | 64 |
| Aspidorhynchidae | 0.259 | 1 | 65 |
| Pholidophoriformes | 0.227 | 1 | 65 |
| Leptolepidae | 0.212 | 1 | 67 |
| Hiodontidae | 0.182 | 1 | 69 |
| Notopteridae | 0.191 | 1 | 70 |
| *Arapaima gigas* | 0.237 | 1 | 71 |
| *Heterotis niloticus* | 0.247 | 1 | 71 |
| Osteoglossinae | 0.184 | 1 | 71 |
| *Pantodon buchholzi* | 0.209 | 1 | 72 |
| Mormyridae | 0.204 | 1 | 72 |
| Gymnarchidae | 0.160 | 1 | 73 |
| *Denticeps clupeoides* | 0.182 | 1 | 75 |
| Clupeidae | 0.237 | 1 | 76 |
| Engraulidae | 0.228 | 1 | 77 |
| Chirocentridae | 0.143 | 1 | 77 |
| Elopidae | 0.183 | 1 | 79 |
| Megalopidae | 0.200 | 1 | 80 |
| Albulinae | 0.226 | 1 | 81 |
| Pterothrissinae | 0.224 | 1 | 82 |
| *Anguilla luzonensis* | 0.136 | 2 | 83 |
| *Moringua ferruginea* | 0.067 | 3 | 84 |
| *Kaupichthys altronasus* | 0.132 | 3 | 84 |
| *Gymnothorax melanosomatus* | 0.097 | 4 | 84 |
| Synaphobranchinae | 0.114 | 1 | 84 |
| *Conger cinereus* | 0.125 | 3 | 88 |
| *Muraenesox bagio* | 0.120 | 5 | 88 |
| *Nettenchelys gephyra* | 0.117 | 6 | 89 |
| *Serrivomer beanii* | 0.140 | 7 | 89 |
| *Luthulenchelys heemstraorum* | 0.072 | 8 | 90 |
| *Saccopharynx berteli* | 0.046 | 9 | 91 |
| Halosauridae | 0.165 | 1 | 92 |
| Lipogenyidae | 0.122 | 1 | 93 |
| Notacanthidae | 0.146 | 1 | 93 |
| *Esox* | 0.266 | 1 | 94 |
| *Dallia pectoralis* | 0.224 | 1 | 95 |
| *Umbra* | 0.242 | 1 | 96 |
| Coregoninae | 0.183 | 1 | 97 |
| Thymallinae | 0.166 | 1 | 98 |
| Salmoninae | 0.259 | 1 | 99 |
| Retropinnidae | 0.190 | 1 | 101 |
| Aplochitoninae | 0.194 | 1 | 102 |
| Galaxiidae | 0.205 | 1 | 102 |
| Osmeridae | 0.207 | 1 | 103 |
| *Plecoglossus altivelis* | 0.191 | 1 | 104 |
| Salangidae | 0.164 | 1 | 104 |
| Argentininae | 0.243 | 1 | 105 |
| *Xenophthalmichthys* | 0.129 | 1 | 105 |
| *Bathylagus* | 0.218 | 1 | 105 |
| Microstomatinae | 0.178 | 1 | 105 |
| Opisthoproctidae | 0.361 | 1 | 106 |
| Alepocephalinae | 0.297 | 1 | 106 |
| Bathylaconinae | 0.237 | 1 | 107 |
| Searsidae | 0.268 | 1 | 107 |
| Gonostomatidae | 0.215 | 1 | 108 |
| Sternoptychidae | 0.309 | 1 | 109 |
| *Chauliodus* | 0.140 | 1 | 109 |
| Stomiatidae | 0.079 | 1 | 110 |
| Astronesthidae | 0.184 | 1 | 110 |
| Melanostomiatidae | 0.116 | 1 | 110 |
| Malacosteidae | 0.266 | 1 | 111 |
| *Idiacanthus* | 0.071 | 1 | 111 |
| Giganturidae | 0.195 | 1 | 111 |
| Chanidae | 0.176 | 1 | 112 |
| Kneriidae | 0.110 | 1 | 113 |
| *Phractolaemus ansorgei* | 0.106 | 1 | 113 |
| *Gonorynchus gonorynchus* | 0.151 | 1 | 114 |
| Characidae | 0.193 | 1 | 116 |
| Erythrinidae | 0.226 | 1 | 117 |
| Ctenoluciidae | 0.287 | 1 | 117 |
| Hepsetidae | 0.259 | 1 | 117 |
| Cynodontidae | 0.178 | 1 | 118 |
| Lebiasininae | 0.189 | 1 | 118 |
| Pyrrhulininae | 0.157 | 1 | 118 |
| Parodontidae | 0.207 | 1 | 119 |
| Gasteropelicidae | 0.170 | 1 | 119 |
| Prochilodontidae | 0.243 | 1 | 120 |
| Curimatidae | 0.192 | 1 | 120 |
| Anostomidae | 0.199 | 1 | 120 |
| Hemiodontidae | 0.188 | 1 | 121 |
| Chilodontidae | 0.186 | 1 | 121 |
| Distichodontidae | 0.229 | 1 | 122 |
| Citharinidae | 0.229 | 1 | 122 |
| Ichthyboridae | 0.223 | 1 | 122 |
| Gymnotidae | 0.131 | 1 | 123 |
| Electrophoridae | 0.127 | 1 | 123 |
| Apteronotidae | 0.119 | 1 | 123 |
| Rhamphichthyidae | 0.137 | 1 | 124 |
| Cyprinidae | 0.219 | 1 | 124 |
| Cyprinidae | 0.193 | 1 | 124 |
| *Myxocyprininus asiaticus* | 0.177 | 1 | 126 |
| *Cycleptus elongatus* | 0.152 | 1 | 126 |
| Ictiobinae | 0.218 | 1 | 127 |
| Catostominae | 0.201 | 1 | 127 |
| Homalopteridae | 0.171 | 1 | 128 |
| Gastromyzontinae | 0.178 | 1 | 128 |
| Cobitidae | 0.173 | 1 | 129 |
| Diplomystidae | 0.176 | 1 | 131 |
| Ictaluridae | 0.248 | 1 | 131 |
| Bagridae | 0.212 | 1 | 132 |
| Cranoglanididae | 0.199 | 1 | 132 |
| Siluridae | 0.159 | 1 | 133 |
| Schilbeidae | 0.181 | 1 | 133 |
| Pangasiidae | 0.137 | 1 | 133 |
| Amblycipitidae | 0.179 | 1 | 134 |
| Amphiliidae | 0.178 | 1 | 134 |
| Akysidae | 0.198 | 1 | 134 |
| Sisoridae | 0.211 | 1 | 135 |
| Clariidae | 0.128 | 1 | 135 |
| *Heteropneustes* | 0.127 | 1 | 136 |
| *Chaca chaca* | 0.272 | 1 | 136 |
| *Olyra* | 0.121 | 1 | 136 |
| *Malapterurus* | 0.179 | 1 | 137 |
| Mochokidae | 0.225 | 1 | 137 |
| Ariidae | 0.239 | 1 | 137 |
| Doradidae | 0.213 | 1 | 138 |
| Auchenipteridae | 0.178 | 1 | 138 |
| bunocephalinae | 0.185 | 1 | 139 |
| Plotosidae | 0.208 | 1 | 139 |
| Pimelodidae | 0.259 | 1 | 140 |
| Pimelodidae | 0.286 | 1 | 140 |
| Ageneiosidae | 0.228 | 1 | 140 |
| *Hypophthalmus edentatus* | 0.245 | 1 | 141 |
| *Helogenes marmoratus* | 0.158 | 1 | 141 |
| Cetopsidae | 0.195 | 1 | 141 |
| Trichomycteridae | 0.146 | 1 | 141 |
| Callichthydae | 0.211 | 1 | 142 |
| Loricariidae | 0.149 | 1 | 142 |
| Astroblepidae | 0.176 | 1 | 143 |
| Aulopodidae | 0.244 | 1 | 144 |
| Synodontidae | 0.226 | 1 | 145 |
| *Harpadon* | 0.173 | 1 | 145 |
| Chlorophthalmidae | 0.246 | 1 | 145 |
| Bathypteroidae | 0.201 | 1 | 146 |
| Ipnopidae | 0.150 | 1 | 146 |
| Scopelosauridae | 0.233 | 1 | 146 |
| Myctophidae | 0.234 | 1 | 147 |
| Neoscopelidae | 0.222 | 1 | 147 |
| Paralepididae | 0.214 | 1 | 148 |
| Omosudidae | 0.257 | 1 | 148 |
| *Alepisaurus* | 0.133 | 1 | 148 |
| *Anotopterus pharao* | 0.296 | 1 | 148 |
| Evermannellidae | 0.191 | 1 | 149 |
| Scopelarchidae | 0.196 | 1 | 149 |
| Polymixiidae | 0.282 | 1 | 150 |
| Percopsidae | 0.244 | 1 | 151 |
| Aphredoderidae | 0.291 | 1 | 151 |
| Amblyopsidae | 0.232 | 1 | 152 |
| Moridae | 0.240 | 1 | 153 |
| *Melanonus* | 0.166 | 1 | 154 |
| *Bregmaceros* | 0.150 | 1 | 154 |
| Gadinae | 0.262 | 1 | 155 |
| Lotinae | 0.198 | 1 | 155 |
| Macruroninae | 0.170 | 1 | 155 |
| *Merluccius* | 0.302 | 1 | 156 |
| Macrouridae | 0.169 | 1 | 156 |
| Brotulinae | 0.160 | 1 | 157 |
| Ophidiinae | 0.177 | 1 | 157 |
| Carapinae | 0.101 | 1 | 158 |
| Zoarcidae | 0.179 | 1 | 158 |
| Batrachoidinae | 0.289 | 1 | 159 |
| Potrichthyinae | 0.255 | 1 | 160 |
| Thalassophryninae | 0.212 | 1 | 161 |
| Lophiidae | 0.282 | 1 | 161 |
| *Brachionichthys* | 0.321 | 1 | 162 |
| Antennariinae | 0.400 | 1 | 163 |
| *Chaunax* | 0.398 | 1 | 163 |
| Ogcocephalidae | 0.441 | 1 | 164 |
| *Caulophryne jordani* | 0.359 | 1 | 165 |
| *Melanocetus* | 0.436 | 1 | 165 |
| Diceratiidae | 0.426 | 1 | 166 |
| *Himantolophus* | 0.483 | 1 | 166 |
| Oneirodidae | 0.438 | 1 | 167 |
| Gigantactinidae | 0.253 | 1 | 167 |
| *Neoceratias spinifer* | 0.284 | 1 | 168 |
| *Centrophryne spinulosa* | 0.383 | 1 | 168 |
| Ceratiidae | 0.327 | 1 | 168 |
| Linophrynidae | 0.510 | 1 | 169 |
| *Indostomus paradoxus* | 0.213 | 1 | 169 |
| Exocoetinae | 0.164 | 1 | 171 |
| Hemiramphinae | 0.190 | 1 | 171 |
| Belonidae | 0.277 | 1 | 172 |
| Scomberesocidae | 0.275 | 1 | 172 |
| *Oryzias* | 0.200 | 1 | 173 |
| Adrianichthydae | 0.312 | 1 | 173 |
| *Horaichthus setnai* | 0.135 | 1 | 173 |
| Cyprinodontidae | 0.249 | 1 | 174 |
| Goodeidae | 0.217 | 1 | 174 |
| *Anableps* | 0.214 | 1 | 175 |
| *Jenynsia* | 0.205 | 1 | 175 |
| Poeciliidae | 0.215 | 1 | 176 |
| Melanotaeniidae | 0.237 | 1 | 177 |
| Atherinidae | 0.197 | 1 | 177 |
| Isonidae | 0.197 | 1 | 178 |
| Neostethidae | 0.200 | 1 | 179 |
| *Lampris guttatus* | 0.268 | 1 | 180 |
| *Velifer* | 0.235 | 1 | 181 |
| *Lophotus* | 0.119 | 1 | 181 |
| Trachipteridae | 0.180 | 1 | 182 |
| *Regalecus* | 0.064 | 1 | 182 |
| *Stylephorus chordatus* | 0.197 | 1 | 183 |
| Ateleopodidae | 0.162 | 1 | 183 |
| *Mirapinna esau* | 0.110 | 1 | 184 |
| Eutaeniophoridae | 0.129 | 1 | 184 |
| Megalomycteridae | 0.165 | 1 | 185 |
| Ctenothrissiformes | 0.192 | 1 | 185 |
| *Monocentris* | 0.368 | 1 | 188 |
| Holocentrinae | 0.313 | 1 | 189 |
| *Parazen pacificus* | 0.311 | 1 | 191 |
| *Macrurocyttus acathopodus* | 0.342 | 1 | 192 |
| Zeidae | 0.315 | 1 | 192 |
| Aulostomidae | 0.287 | 1 | 194 |
| *Fistularia commersonii* | 0.326 | 10 | 195 |
| Macrorhamphosidae | 0.419 | 1 | 195 |
| Centriscidae | 0.335 | 1 | 196 |
| *Solenostomus* | 0.261 | 1 | 196 |
| Syngnathinae | 0.165 | 1 | 197 |
| Aulorhynchidae | 0.249 | 1 | 198 |
| Gasterosteidae | 0.234 | 1 | 198 |
| Alabetidae | 0.134 | 1 | 199 |
| *Synbranchus lampreia* | 0.141 | 11 | 200 |
| Scorpaeninae | 0.314 | 1 | 202 |
| Tetraroginae | 0.301 | 1 | 202 |
| Synanceiidae | 0.377 | 1 | 203 |
| *Mimous* | 0.321 | 1 | 204 |
| Triglidae | 0.262 | 1 | 204 |
| *Caracanthus* | 0.339 | 1 | 205 |
| Aploactininae | 0.320 | 1 | 206 |
| Pataicinae | 0.251 | 1 | 206 |
| *Gnathanacanthus goetzeei* | 0.328 | 1 | 207 |
| Anoplopomatidae | 0.249 | 1 | 207 |
| Hexagramminae | 0.228 | 1 | 208 |
| Zaniolepididae | 0.206 | 1 | 209 |
| Platycephalinae | 0.261 | 1 | 210 |
| *Hoplichthys* | 0.254 | 1 | 210 |
| Congiopodidae | 0.258 | 1 | 210 |
| Icelidae | 0.305 | 1 | 211 |
| Cottidae | 0.332 | 1 | 212 |
| Cottocomephoridae | 0.254 | 1 | 213 |
| *Comephorus* | 0.274 | 1 | 213 |
| Cottunculidae | 0.336 | 1 | 214 |
| Psychrolutidae | 0.305 | 1 | 214 |
| Aspidophoroidinae | 0.176 | 1 | 214 |
| Agoninae | 0.163 | 1 | 215 |
| Cyclopterinae | 0.256 | 1 | 215 |
| Leparinae | 0.221 | 1 | 216 |
| Dactylopteridae | 0.201 | 1 | 216 |
| Pegasidae | 0.332 | 1 | 217 |
| Centropomidae | 0.294 | 1 | 219 |
| Percichthydae | 0.312 | 1 | 220 |
| Serranidae | 0.329 | 1 | 220 |
| Grammistidae | 0.303 | 1 | 221 |
| Pseudochromidae | 0.209 | 1 | 222 |
| Plesiopidae | 0.296 | 1 | 223 |
| Acanthoclinidae | 0.241 | 1 | 224 |
| *Glaucosoma* | 0.307 | 1 | 224 |
| Theraponidae | 0.271 | 1 | 225 |
| Banjosidae | 0.285 | 1 | 225 |
| Kuhliidae | 0.218 | 1 | 226 |
| Centrarchinae | 0.292 | 1 | 227 |
| Centrarchinae | 0.288 | 1 | 227 |
| Apogonidae | 0.314 | 1 | 228 |
| Percinae | 0.267 | 1 | 229 |
| Percinae | 0.220 | 1 | 230 |
| Luciopercinae | 0.253 | 1 | 230 |
| Sillaginidae | 0.221 | 1 | 230 |
| Branchiostegidae | 0.246 | 1 | 231 |
| *Lactarius* | 0.274 | 1 | 231 |
| Pomatomidae | 0.217 | 1 | 232 |
| *Rachycentron canadum* | 0.188 | 1 | 232 |
| Echeneidae | 0.225 | 1 | 232 |
| Carangidae | 0.237 | 1 | 233 |
| Coryphaenidae | 0.152 | 1 | 233 |
| *Formio niger* | 0.210 | 1 | 234 |
| Mene maculata | 0.273 | 1 | 234 |
| Leiognathidae | 0.234 | 1 | 235 |
| Emmelichthydae | 0.238 | 1 | 236 |
| Lutjanidae | 0.283 | 1 | 237 |
| Gerreidae | 0.258 | 1 | 238 |
| Pomadasyidae | 0.310 | 1 | 238 |
| Lethrinidae | 0.260 | 1 | 239 |
| Sparidae | 0.254 | 1 | 240 |
| Sciaenidae | 0.223 | 1 | 240 |
| Mullidae | 0.244 | 1 | 241 |
| Pempheridae | 0.283 | 1 | 241 |
| Ephippidae | 0.231 | 1 | 244 |
| Chaetodontinae | 0.275 | 1 | 245 |
| Pomacanthinae | 0.204 | 1 | 246 |
| *Enoplosus armatus* | 0.240 | 1 | 246 |
| Pentacerotidae | 0.376 | 1 | 247 |
| Nandinae | 0.306 | 1 | 248 |
| Embiotocidae | 0.269 | 1 | 249 |
| Cichlidae | 0.259 | 1 | 250 |
| Pomacentrinae | 0.251 | 1 | 252 |
| *Gadopsis marmoratus* | 0.223 | 1 | 252 |
| Cirrhitidae | 0.294 | 1 | 253 |
| Owstoniidae | 0.186 | 1 | 255 |
| Cepolidae | 0.088 | 1 | 255 |
| Mugilidae | 0.174 | 1 | 256 |
| Sphyraena | 0.255 | 1 | 256 |
| Polynemidae | 0.209 | 1 | 257 |
| Labridae | 0.279 | 1 | 258 |
| Odacidae | 0.255 | 1 | 258 |
| Scaridae | 0.255 | 1 | 259 |
| Trichodontidae | 0.218 | 1 | 260 |
| Ophistognathidae | 0.221 | 1 | 260 |
| *Champsodon* | 0.247 | 1 | 260 |
| Chiasmodontidae | 0.254 | 1 | 261 |
| Bathymasteridae | 0.278 | 1 | 261 |
| Mugiloididae | 0.238 | 1 | 262 |
| Cheimarrhichthyidae | 0.250 | 1 | 263 |
| *Trachinus* | 0.216 | 1 | 264 |
| Uranoscopidae | 0.261 | 1 | 265 |
| Leptoscopidae | 0.225 | 1 | 265 |
| Dactyloscopidae | 0.252 | 1 | 265 |
| Channichthyidae | 0.351 | 1 | 267 |
| Congrogadidae | 0.155 | 1 | 268 |
| *Notograptus* | 0.135 | 12 | 269 |
| Peronedysidae | 0.168 | 1 | 269 |
| Ophiclinidae | 0.172 | 1 | 269 |
| Triperygiidae | 0.243 | 1 | 270 |
| Clinidae | 0.254 | 1 | 270 |
| Blenniidae | 0.206 | 1 | 271 |
| Stichaeidae | 0.193 | 1 | 272 |
| *Zaprora silenus* | 0.190 | 1 | 275 |
| Scytalina cerdale | 0.120 | 1 | 275 |
| *Icosteus aenigmaticus* | 0.208 | 1 | 276 |
| Ammodytidae | 0.134 | 1 | 276 |
| Eleotridae | 0.255 | 1 | 277 |
| Gobiidae | 0.194 | 1 | 278 |
| *Rhyacichthys* | 0.237 | 1 | 279 |
| Gobioididae | 0.107 | 1 | 280 |
| Trypauchenidae | 0.167 | 1 | 280 |
| Microdesmidae | 0.139 | 1 | 281 |
| Kurtidae | 0.252 | 1 | 281 |
| Acanthurinae | 0.249 | 1 | 282 |
| *Zanclus* | 0.320 | 1 | 283 |
| Siganidae | 0.170 | 1 | 283 |
| Trichiuridae | 0.202 | 1 | 284 |
| Scombridae | 0.213 | 1 | 285 |
| *Xiphias gladius* | 0.458 | 1 | 286 |
| Istiophoridae | 0.349 | 1 | 286 |
| *Amarsipus carlsbergi* | 0.233 | 1 | 287 |
| Centrolophidae | 0.204 | 1 | 288 |
| Nomeidae | 0.260 | 1 | 288 |
| *Ariomma* | 0.254 | 1 | 289 |
| *Tetragonurus* | 0.205 | 1 | 289 |
| Stromateidae | 0.217 | 1 | 289 |
| Macropodinae | 0.243 | 1 | 291 |
| Grichogasterinae | 0.234 | 1 | 291 |
| *Luciocephalus pulcher* | 0.353 | 1 | 292 |
| Channidae | 0.277 | 1 | 293 |
| Mastacembelidae | 0.159 | 1 | 293 |
| Gobiesocidae | 0.271 | 1 | 294 |
| Callionymidae | 0.231 | 1 | 295 |
| Psettodidae | 0.218 | 1 | 296 |
| Citharinae | 0.270 | 1 | 297 |
| Paralichthynae | 0.221 | 1 | 298 |
| Pleuronectini | 0.215 | 1 | 299 |
| Samarinae | 0.151 | 1 | 299 |
| Soleinae | 0.169 | 1 | 300 |
| Cynoglossinae | 0.176 | 1 | 301 |
| *Symphurus* | 0.188 | 1 | 301 |
| Triacanthodidae | 0.282 | 1 | 302 |
| Balistinae | 0.253 | 1 | 303 |
| Ostraciontinae | 0.159 | 1 | 304 |
| Tetraodontinae | 0.296 | 1 | 305 |
| Diodontidae | 0.323 | 1 | 306 |
| Molidae | 0.286 | 1 | 307 |

References:

1: Nelson, J.S. 1994. *Fishes of the world. Third Edition*. John Wiley & Sons, Inc., New York.

2: Watanabe, S., Aoyama, J., Tsukamoto, K. 2009. A new species of freshwater eel Anguilla luzonensis (Teleostei: Anguillidae) from Luzon Island of the Phillipines. *Fish Sci.* **75**: 387-392.

3: Randall, J.E., Allen, G.R. & Steene, R.C. 1997. *Fishes of the Great Barrier Reef and Coral Sea*. University of Hawaii Press, Honolulu.

4: Loh, K.-H., Shao, K.-T. & Chen, H.-M. 2011. *Gymnothorax melanosomatus*, a new moray eel (Teleostei: Anguilliformes: Muraenidae) from southeastern Taiwan. *Zootaxa* **3134**: 43-52.

5: Lin, J., Shao, K.-T. & Chen, H.-M. (2013). Taxonomic study of pike congers (Anguilliformes: Mureanesocidae) with identification of mureanesocid collections in Taiwan. *J. Mar. Sci. Tech.* **21**: 166-174.

6: Smith, D.G., Lin, J., Chen, H.-M. & Pogonoski, J.J. 2015. The eels of the genus *Nettenchelys*, with description of a new species from Taiwan (Teleostei: Anguilliformes, Nettastomatidae). *Zootaxa* 4060: 105-120.

7: McEachran, J.D. & Fechhelm, J.D. 1998. *Fishes of the Gulf of Mexico, Vol. 1 Myxiniformes to Gasterosteiformes*. University of Texas Press, Austin.

8: McCosker, J.E. 2007. *Luthulenchelys heemstraorum*, a new genus and species of snake eel (Anguilliformes: Ophichthidae) from KwaZulu-Natal, with comments on *Ophichthus rutidoderma* (Bleeker, 1853) and its synonyms. *Smithiana Bull.* **7**: 3-7.

9: Tighe, K.A. & Nielsen, J.G. 2000. Saccopharynx berteli, a new gulper eel from the Pacific Ocean (Teleostei, Saccopharyngidae). Ichthyol. Res. 47: 39-41.

10: Meloni, D. & Piras, P. 2013. New record of the blue-spotted cornetfish, *Fistularia commersonii* Rüppell, 1838 (Synghnathiformes Fistularidae), in the South-Western Mediterranean Sea. Biodiversity Journal 4: 435-438.

11: Favorito, S.E., Zanata, A.M. & Assumpção, M.I. 2005. A new *Synbranchus* (Teleostei: Synbranchiformes: Synbranchidae) from ilha de Marajo, Para, Brazil, with notes on its reproductive biology and larval development. *Neotrop. Ichthyol.* **3**: 319-328.

12: Mooi, R.D. & Gill, A.C. 2004. Notograptidae, sister to Acanthoplesiops Regan (Teleostei: Plesiopidae: Acanthoclininae), with comments on biogeography, diet and morphological convergence with Congrogadinae (Teleostei: Pseudochromidae). Zool. J. Linn. Soc. 141: 179-205.
