# Supplementary material for: Terrestrial capture of prey by the reedfish, a model species for stem tetrapods
Source: Ecol Evol. 2017 Apr 21;7(11):3856–60. doi: 10.1002/ece3.2694 (PMC5468123; doi:10.1002/ece3.2694)
Supplement: Supplementary file 2 [file ECE3-7-3856-s002.docx]

**Supplementary Table S2:** List of the head length (HL) to total length (TL) ratios for species of Actinopterygii that capture prey on land as displayed in Figure 2 (middle panels), and their literature sources.

| **species name** | **HL / TL** | **Reference** |
| --- | --- | --- |
| *Erpetoichtys calabaricus* | 0.0797 | 1 |
| *Erpetoichtys calabaricus* | 0.0735 | 1 |
| *Erpetoichtys calabaricus* | 0.0715 | 1 |
| *Erpetoichtys calabaricus* | 0.0826 | 1 |
| *Erpetoichtys calabaricus* | 0.0849 | 1 |
| *Erpetoichtys calabaricus* | 0.075 | 1 |
| *Channallabes apus* | 0.0947 | 2 |
| *Channallabes apus* | 0.074 | 3 |
| *Channallabes apus* | 0.0723 | 3 |
| *Gymnallabes typus* | 0.1074 | 3 |
| *Gymnallabes typus* | 0.0996 | 3 |
| *Gymnallabes typus* | 0.1174 | 3 |
| *Gymnallabes typus* | 0.1112 | 3 |
| *Gymnallabes typus* | 0.1018 | 3 |
| *Anableps anableps* | 0.2124 | 4 |
| *Anableps anableps* | 0.1701 | 4 |
| *Anableps anableps* | 0.1852 | 4 |
| *Periophthalmus barbarus* | 0.2167 | 5 |
| *Periophthalmus barbarus* | 0.2294 | 5 |
| *Periophthalmus barbarus* | 0.2558 | 5 |
| *Periophthalmus barbarus* | 0.2257 | 5 |

References:

1: This study

2: Devaere, S., Adriaens, D., Verraes, W. & Teugels, G.G. 2001. Cranial morphology of the anguilliform clariid Channallabes apus (Günther, 1873) (Teleostei: Siluriformes): are adaptations related to powerful biting? *J. Zool., Lond.* **255**: 235-250.

3: Cabuy, E., Adriaens, D., Verraes, W. & Teugels, G.G. 1999. Comparative study on the cranial morphology of *Gymnallabes typus* (Siluriformes: Clariidae) and their less anguilliform relatives, *Clariallabes melas* and *Clarias gariepinus*. *J. Morphol.* **240**: 169-194.

4: Michel, K.B., Aerts, P., Gibb, A.C. & Van Wassenbergh, S. 2015b. Functional morphology and kinematics of terrestrial feeding in the largescale foureyes (*Anableps anableps*). *J. Exp. Biol.* **218**: 2951-2960.

5: Michel, K.B., Heiss, E., Aerts, P. & Van Wassenbergh, S. 2015a. A fish that uses its hydrodynamic tongue to feed on land. *Proc. R. Soc. B* **282**: 20150057.
