# Supplementary material for: Terrestrial capture of prey by the reedfish, a model species for stem tetrapods
Source: Ecol Evol. 2017 Apr 21;7(11):3856–60. doi: 10.1002/ece3.2694 (PMC5468123; doi:10.1002/ece3.2694)
Supplement: Supplementary file 3 [file ECE3-7-3856-s003.docx]

**Supplementary Table S3:** List of the head length (HL) to total length (TL) ratios for fossil sarcopterygians as displayed in Figure 2 (bottom panel), and their literature sources.

| **species name** | **HL / TL** | **Reference** |
| --- | --- | --- |
| *Gooloogongia loomesi* | 0.2021 | 1 |
| *Eusthenopteron* | 0.2168 | 2 |
| *Ichthyostega* | 0.1875 | 3 |
| *Acanthostega* | 0.1906 | 3 |

References:

1: Johanson, Z. & Ahlberg, P.E. 1998. A complete primitive rhizodont from Australia. *Nature* **394**: 569-573.

2: Ahlberg, P.E. & Milner, A.R. 1994. The origin and early diversification of tetrapods. *Nature* **368**: 507-514.

3: Ahlberg, P.E., Clack, J.A. & Blom, H. 2005. The axial skeleton of the Devonian tetrapod Ichthyostega. *Nature* **437**: 137-140.
